# Characterization of Circulating Protein Profiles in Individuals with Prader–Willi Syndrome and Individuals with Non-Syndromic Obesity

**DOI:** 10.3390/jcm13195697

**Published:** 2024-09-25

**Authors:** Devis Pascut, Pablo José Giraudi, Cristina Banfi, Stefania Ghilardi, Claudio Tiribelli, Adele Bondesan, Diana Caroli, Graziano Grugni, Alessandro Sartorio

**Affiliations:** 1 Fondazione Italiana Fegato—ONLUS, Liver Cancer Unit, 34149 Trieste, Italy; ctliver@fegato.it; 2 Fondazione Italiana Fegato—ONLUS, Metabolic Liver Disease Unit, 34149 Trieste, Italy; 3 Unit of Functional Proteomics, Metabolomics, and Network Analysis, Centro Cardiologico Monzino, IRCCS, 20138 Milan, Italy; cristina.banfi@cardiologicomonzino.it (C.B.);; 4 Istituto Auxologico Italiano, IRCCS, Experimental Laboratory for Auxo-Endocrinological Research, 28824 Piancavallo-Verbania, Italy; a.bondesan@auxologico.it (A.B.); g.grugni@auxologico.it (G.G.); sartorio@auxologico.it (A.S.)

**Keywords:** proteome, circulating biomarkers, Prader–Willi syndrome, non-syndromic obesity, neuromodulatory factors

## Abstract

**Background:** Prader–Willi syndrome (PWS) is a rare genetic disorder characterized by distinctive physical, cognitive, and behavioral manifestations, coupled with profound alterations in appetite regulation, leading to severe obesity and metabolic dysregulation. These clinical features arise from disruptions in neurodevelopment and neuroendocrine regulation, yet the molecular intricacies of PWS remain incompletely understood. **Methods**: This study aimed to comprehensively profile circulating neuromodulatory factors in the serum of 53 subjects with PWS and 34 patients with non-syndromic obesity, utilizing a proximity extension assay with the Olink Target 96 neuro-exploratory and neurology panels. The ANOVA *p*-values were adjusted for multiple testing using the Benjamani–Hochberg method. Protein–protein interaction networks were generated in STRING V.12. Corrplots were calculated with R4.2.2 by using the Hmisc, Performance Analytics, and Corrplot packages **Results**: Our investigation explored the potential genetic underpinnings of the circulating protein signature observed in PWS, revealing intricate connections between genes in the PWS critical region and the identified circulating proteins associated with impaired oxytocin, NAD metabolism, and sex-related neuromuscular impairment involving, CD38, KYNU, NPM1, NMNAT1, WFIKKN1, and GDF-8/MSTN. The downregulation of CD38 in individuals with PWS (*p* < 0.01) indicates dysregulation of oxytocin release, implicating pathways associated with NAD metabolism in which KYNU and NMNAT1 are involved and significantly downregulated in PWS (*p* < 0.01 and *p* < 0.05, respectively). Sex-related differences in the circulatory levels of WFIKKN1 and GDF-8/MSTN (*p* < 0.05) were also observed. **Conclusions:** This study highlights potential circulating protein biomarkers associated with impaired oxytocin, NAD metabolism, and sex-related neuromuscular impairment in PWS individuals with potential clinical implications.

## 1. Introduction

Prader–Willi syndrome (PWS) is a rare genetic disorder, occurring in about 1 in 21,000 newborns [1], known for its distinctive physical, cognitive, and behavioral manifestations. It results from a lack of expression of genes located on the paternal chromosome 15q11.2-q13, leading to disruptions in neurodevelopment and neuroendocrine regulation. The complex clinical phenotype observed in individuals with PWS is driven by various genetic mechanisms, including interstitial deletion of the paternal chromosome 15 (del15q11.2-q13) (DEL15), maternal uniparental disomy of chromosome 15 (UPD15), and imprinting defects [2]. The most common genetic abnormality observed in PWS, occurring in about 60–70% of cases, is DEL15. UPD15 accounts for 25–35% of cases, while imprinting defects contribute to 1–4% of cases [3]. Clinically, PWS is characterized by severe neonatal hypotonia, poor feeding, and a lack of appetite in infancy, followed by morbid obesity (if uncontrolled), making it the most common syndromic form of life-threatening obesity [4]. The development of obesity arises from hyperphagic behavior and dysfunctional appetite control mechanisms [5,6,7]. Endocrine abnormalities such as hypogonadism, growth hormone deficiency, central hypothyroidism, adrenal insufficiency, premature adrenarche, and osteoporosis are common in individuals with PWS [8,9,10]. Moreover, developmental delays, intellectual disabilities, speech and articulation defects, as well as behavioral and psychiatric features, including tantrums, obsessive compulsive tendencies, and autistic-like traits, are prevalent and can be associated with specific genetic subtypes [8]. The paternally expressed genes, particularly small nuclear ribonucleoprotein polypeptide N (*SNRPN*) [9] and MAGE family member L2 (*MAGEL2*), are extensively studied for their roles in neurodevelopment and synaptic function [10]. Other genes include Necdin-MAGE Family Member (*NDN*), Makorin ring finger protein 3 (*MKRN3*), and Nuclear pore-associated protein 1 *(NPAP1*) [11,12]. The same chromosomal region contains several ncRNA genes, such as multiple clusters of snoRNAs, such as *SNORD116*, and the long non-coding RNAs Prader–Willi/Angelman Region RNA 1 (*PWAR1* or *PAR-1*), Prader–Willi/Angelman Region RNA 2 (*PWAR1* or *PAR-2*), and Imprinted in Prader–Willi (*IPW*) [13,14,15,16,17]. Dysregulation of these genes contributes to the typical phenotype in PWS [3]. For example, the absence of the *NDN* gene has been suggested to contribute to the hypogonadotropic hypogonadal phenotype [18], to regulate the proliferation of white adipocyte progenitor cells in mice [19], and to determine both heart systolic and diastolic dysfunction in mice [20]. Similarly, the loss of *Magel2* has been shown to reduce fertility [21] and determine hypothalamic endocrine dysfunction in mice [22]. Moreover, *Snord116 +/−* mice exhibit cognitive dysfunction [23] and the deletion of *Snord116* in the mediobasal hypothalamus of adult mice leads to a hyperphagic phenotype, with some animals developing obesity [24]. Molecular alterations in PWS extend beyond the function of individual genes within the PWS locus. For example, *SNRPN* encodes a component of the small nuclear ribonucleoprotein complex, responsible for pre-mRNA processing and contributing to tissue-specific alternative splicing of several target mRNAs [25,26,27]. For example, the knockdown of *Snrpn*, in cultured primary mice cortical neurons, affected the expression of nuclear receptor subfamily 4 group A member 1 (*Nr4a1*) [28], a gene with crucial functions in neural development and plasticity [29]. Additionally, *SNORD116* deletion impaired neuronal differentiation, proliferation, and survival, possibly due to the impact on multiple mRNA targets [30]. The emerging picture describes the genes of the PWS locus as part of a complex regulatory network that likely orchestrates a cascade of events leading to a broad spectrum of endocrinological, neurological, and psychological complications in PWS. These genetic perturbations may influence the circulating proteome in PWS, potentially revealing circulating proteins regulated by genes in the critical region of the disease. Investigating these alterations in the circulatory proteome could provide new insights into the clinical aspects of PWS and identify easily accessible biomarkers to monitor disease complications and treatment efficacy.

## 2. Materials and Methods

### 2.1. Patients

The study recruited 53 individuals with PWS (29 females and 24 males, mean age ± SD: 35.5 ± 11 yrs, BMI: 38.6 ± 8.9 kg/m^2^) and 34 patients with non-syndromic obesity (OB) (16 females and 18 males, mean age ± SD: 35.2 ± 9.2 yrs, BMI: 41.1 ± 4.4 kg/m^2^). The inclusion criteria were: (1) genetically confirmed diagnosis for PWS subjects; (2) individuals of both sexes, aged > 18 years; (3) BMI > 25 kg/m^2^ for subjects with PWS and BMI > 30 kg/m^2^ for subjects with non-syndromic obesity; and (4) Caucasian origin. Exclusion criteria were: (1) secondary obesity, including genetic (e.g., monogenic and other syndromic conditions other than PWS), organic (e.g., kidney or liver diseases, cerebral neoplasms), endocrine, (e.g., uncontrolled hypothyroidism, endogenous hypercortisolism, having being excluded by standard endocrine assessments), or iatrogenic forms (e.g., chronic exposure to glucocorticoids); (2) individuals (and/or their parents) who refused to sign the consent form. All the enrolled patients were hospitalized for a multidisciplinary 3-week body weight reduction program at the Istituto Auxologico Italiano, IRCCS (Piancavallo (VB), Italy, at the Division of Auxology (subjects with PWS) and the Division of Metabolic Diseases (subjects with non-syndromic obesity). All subjects with PWS showed the typical clinical phenotype [3]. Cytogenetic analysis was performed in all patients with PWS, including the Methylation-Specific Multiplex Ligation-dependent Prob Amplification (MS-MLPA) and microsatellite analysis, when appropriate. Forty-one subjects had DEL15, and ten patients had UPD15, while a positive methylation test was demonstrated in the remaining two PWS cases (males), but the underlying genetic defect was not identified. The physical assessment comprised determining height, weight, waist, and hip circumference under fasting conditions and post voiding. Standing height was ascertained using a wall-mounted Harpenden Stadiometer (Holtain Limited, Crymych, UK). Subjects were weighed with minimal clothing, rounded to the nearest 0.1 kg, employing standard equipment. Obesity was defined using a BMI threshold of 30 kg/m^2^. Waist and hip circumferences were measured with a flexible tape measure while subjects stood erect, arms at their sides, and feet together. Waist circumference was measured midway between the iliac crest and last rib, while hip circumference was measured at the widest part of the hip, level with the greater trochanter. Fat-free mass (FFM) and fat mass (FM) were evaluated via bioimpedance analysis using the Human-IM Touch device (DS-Medigroup, Milan, Italy). Measurements were performed according to the method described by Lukaski [31] after 20 min of resting in a supine position with arms and legs relaxed and not in contact with other body parts. FFM was calculated using the prediction equation [32], and FM was derived as the difference between BM and FFM. Diastolic and systolic blood pressure was measured twice in the supine position after 5 min of rest on the dominant arm with an aneroid sphygmomanometer (TemaCertus, Milan, Italy), with a 3 min interval between readings. Appropriate cuff sizes were used, and mean values were calculated, rounded to the nearest five mmHg. The average of three measurements on different days was used. Treatments and other comorbidities present at enrollment are reported in Table S1. The Ethics Committee of Istituto Auxologico Italiano Milan, Italy (ethical committee code: CE: 2022_03_15_07), approved the study. All procedures in the study complied with the Helsinki Declaration of 1975, as revised in 2008. The research procedure was explained to each participant, and written informed consent was obtained from the subjects and their parents when it was appropriate (i.e., subjects with PWS).

### 2.2. Biochemical Parameters

Total cholesterol (T-C), high-density lipoprotein cholesterol (HDL-C), low-density lipoprotein cholesterol (LDL-C), triglycerides (TG), glucose, insulin, alanine aminotransferase (ALT), and aspartate aminotransferase (AST) were measured using standard enzymatic methods (Roche Diagnostics, Mannheim, Germany). Glycated hemoglobin (HbA1c) was measured with capillary electrophoresis (Capillarys 2 Flex Piercing, Sebia, Lisses, France).

For each patient, the homeostatic model assessment of insulin resistance (HOMA-IR) was also calculated using the following formula: (Insulin [μU/mL] × glucose [mmol/L])/22.5 [33,34].

### 2.3. Serum Collection for Proteomics

Following an overnight fasting period, blood samples were collected via standard venipuncture using BD Vacutainer^®^ serum separating tubes (BD—Plymouth, UK). The tubes were centrifuged at 1900× *g* for 10 min at 4 °C. After the initial centrifugation, the resulting supernatants were carefully transferred into fresh tubes. Subsequently, samples underwent a second centrifugation step at 16,000× *g* for 10 min at 4 °C. Supernatants were divided into new tubes and promptly frozen at −80 °C to ensure long-term storage until further analysis.

### 2.4. Circulating Proteome Profiling and Analysis

Serum samples underwent analysis utilizing the proximity extension assay (PEA) with Olink Target 96 neuro-exploratory and neurology panels (Olink Proteomics, Uppsala, Sweden), which enable multiplex detection of circulating proteins. These two panels were chosen due to the disruptions in neurodevelopment and neuroendocrine regulation in Prader–Willi syndrome (PWS), which lead to multiple clinical manifestations. In PEA, target proteins specifically bind to double oligonucleotide-labeled antibody probes. Subsequently, microfluidic real-time PCR amplifies the oligonucleotide sequence for quantitative DNA sequence detection. Quality control (QC) and normalization were applied to the threshold cycle (Ct) data obtained from the internal and external controls. QC was conducted to ensure the accuracy and reliability of the data. Four internal controls were added to each sample to monitor both assay performance and individual sample quality. QC was performed in two steps. First, each sample plate was evaluated based on the standard deviation of the internal controls, with a threshold of less than 0.2 NPX. Only data from plates that met this criterion were included in the analysis. Second, the quality of each individual sample was assessed by measuring the deviation of the internal controls from the median value. Samples with a deviation of less than 0.3 NPX from the median passed QC. Protein levels were quantified on a relative scale and expressed as normalized protein expression (NPX) units, presented in a logarithmic scale (log2). Higher NPX values indicate higher protein concentrations. The list of proteins analyzed is detailed in File S1.

Data visualization and exploration and the initial statistical analysis were conducted using the Olink Statistical Analysis web-based app. The NPX dataset was uploaded, and samples failing quality controls were excluded. The results were presented as NPX median values and inter-quartile range (IQR) for each marker within the sample group unless otherwise specified. The reported p-values from the ANOVA analysis were adjusted for multiple testing using the Benjamani–Hochberg method.

### 2.5. Bioinformatics Analysis

We performed gene mapping and enrichment analyses on the Gene Ontology (GO) (http://geneontology.org/) database using the Metascape web tool v3.5.20240101 (https://metascape.org/gp/index.html#/main/, accessed on 1 June 2024) [35] or g: Profiler [36] (https://biit.cs.ut.ee/gprofiler/page/docs, accessed on 1 June 2024). The enrichment analysis was conducted using all genes in the genome as the background. Terms meeting the criteria of a *p*-value < 0.01, a minimum count of 3, and an enrichment factor > 1.5 were collected. The enrichment factor represents the ratio between the observed and expected counts by chance. These terms were then grouped into clusters based on their membership similarities. The *p*-values were calculated using the cumulative hypergeometric distribution, while the q-values were computed via the Benjamani–Hochberg procedure to correct for multiple testing. Hierarchical clustering was performed on the enriched terms using Kappa scores as the similarity metric. Sub-trees with a similarity score exceeding 0.3 were considered a cluster. The protein list was mapped within the DisGeNET database (http://www.disgenet.org) [37], collecting genes and variants associated with human diseases. Protein–protein interaction analysis was conducted for each given protein using STRING V.12 (https://string-db.org/) [38]. To identify genes and proteins consistently regulated in different studies, datasets were analyzed by the InteractiVenn web-based tool [39] and intersections were visualized through a Venn diagram. Correlation matrices were generated with R 4.2.2 by using the Hmisc (version 5.0-1) [40], Performance Analytics (version 2.0.4) [41], and Corrplot (version 0.92) [42] packages.

### 2.6. Statistical Methods

To determine significant differences among continuous variables with normal distributions, we employed the *t*-test, while the Mann–Whitney test was utilized for non-normally distributed variables. The D’Agostino omnibus test was used to test the normality distribution of variables. Categorical variables were assessed using the chi-square test. For multiple comparisons, the Kruskal–Wallis test within the framework of the one-way ANOVA procedure was applied, with correction using the Benjamani–Hochberg method.

## 3. Results

### 3.1. Characteristics of the Participants

Table 1 shows the clinical characteristics of the participants. Significant differences (*p* < 0.05) between groups were observed for FM, insulin, HOMA index, and HDL cholesterol. All these parameters, except for HDL cholesterol, were higher in subjects with non-syndromic obesity (OB) compared to subjects with Prader–Willi syndrome (PWS) (Table 1).

### 3.2. Differences in the Circulating Proteome in PWS: Identification of Gender-Associated Protein Markers

**Figure 1:**
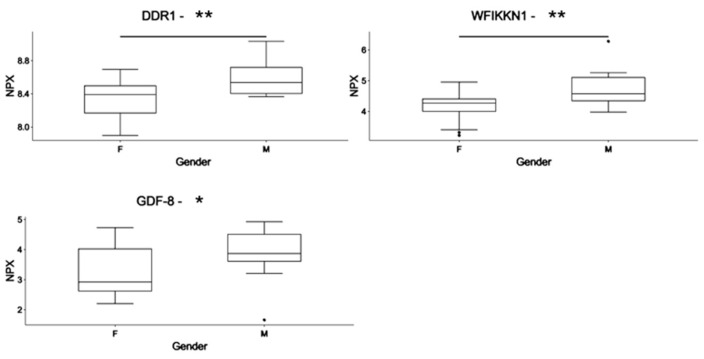
Differences in the circulating protein biomarkers according to sex in the PWS subjects. Differences in the expression of protein markers are expressed as a normalized protein expression (NPX) unit. Values are presented as median with their respective interquartile ranges. Statistical significance levels are denoted as follows: *, *p* < 0.05; **, *p* < 0.01. DDR1: Epithelial discoidin domain-containing receptor 1; WFIKKN1: WAP Kazal immunoglobulin Kunitz and NTR domain-containing protein 1; GDF-8: Growth/Differentiation Factor 8, also known as myostatin; F: female (*n* = 29); M: male (*n* = 24).

The circulating proteome was investigated by using the Olink neurology and neuro-exploratory panels consisting of 92 different human protein biomarkers each (File S1). To identify the influence of sex in circulating protein expression, we compared the circulating proteome profiles of males to those of females. Epithelial discoidin domain-containing receptor 1 (DDR1), WAP Kazal immunoglobulin Kunitz and NTR domain-containing protein 1 (WFIKKN1), and Growth/Differentiation Factor 8 (GDF-8), also known as myostatin, were significantly lower in female subjects with PWS (*n* = 29) compared to males (*n* = 24) (*p* < 0.05) (Table 2, and Figure 1). No differences were observed between males and females in the OB group (*n* = 18 vs. 16, respectively). We also investigated the presence of possible variations in the circulating protein expression according to the type of chromosomal alteration present in PWS. No differences were observed when comparing PWS with del15 and PWS with UPD.

### 3.3. Differences in Circulating Protein Markers between Subjects with PWS and Subjects with Non-Syndromic Obesity

**Figure 2:**
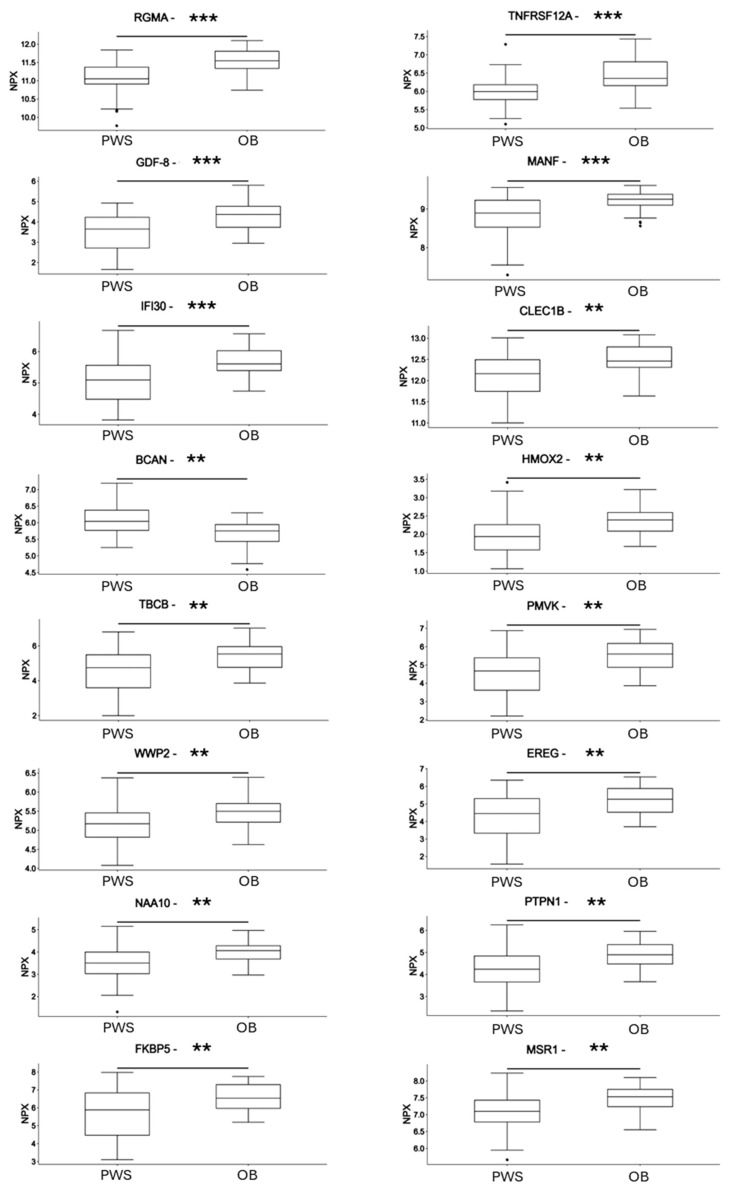

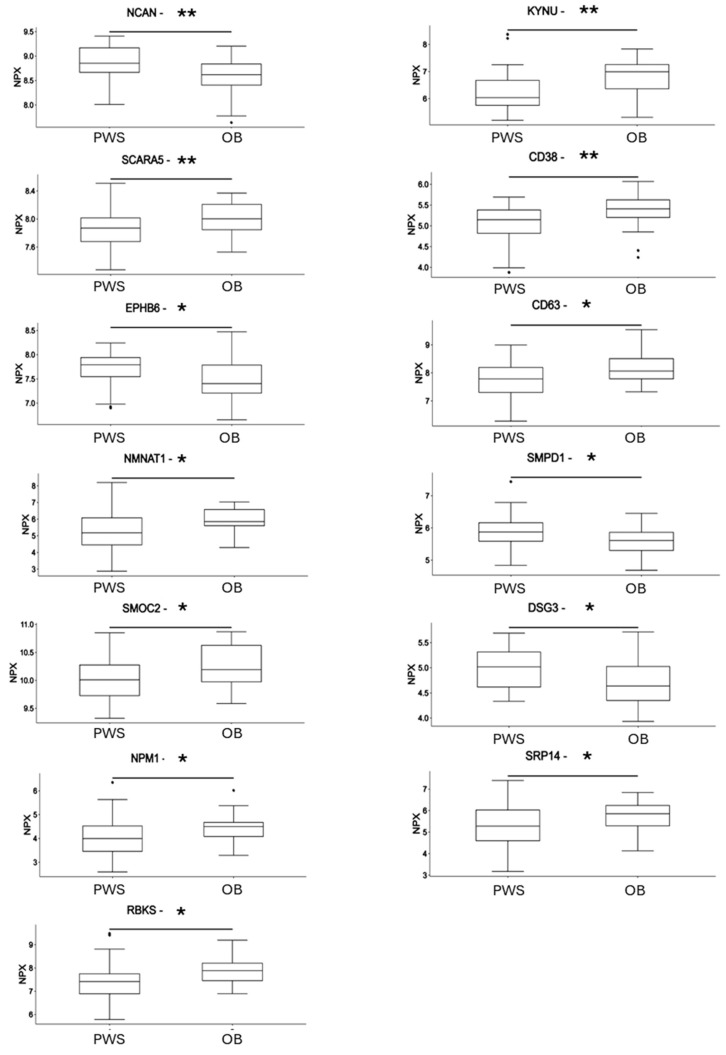
Differences in the circulating protein biomarkers between the subjects with PWS and subjects with non-syndromic obesity. Differences in the expression of protein markers are expressed as a normalized protein expression (NPX) unit. Values are presented as median with their respective interquartile ranges. Statistical significance levels are as follows: *, *p* < 0.05; **, *p* < 0.01; ***, *p* < 0.001. PWS: subjects with Prader–Willi syndrome; OB: subjects with non-syndromic obesity.

The targeted proteomic analysis of serum derived from PWS and OB subjects revealed a total of 29 proteins with a significant differential expression between the two groups of patients (Table 3, Figure 2). Most of the circulating proteins showed significant downregulation in the PWS group (Figure 2) with Tubulin Folding Cofactor B (TBCB) and Phosphomevalonate Kinase (PMVK) showing the highest reduction in PWS (FC = 0.55). Five proteins, Brevican (BCAN), Neurocan (NCAN), Ephrin type-B receptor 6 (EPHB6), Sphingomyelin phosphodiesterase 1 (SMPD1), and Desmoglein 3 (DSG3), were upregulated in the serum of PWS subjects (Table 3 and Figure 2).

### 3.4. Exploring the Influence of Imprinted Genes on Circulating Protein Signatures in Prader–Willi Syndrome

**Figure 3:**
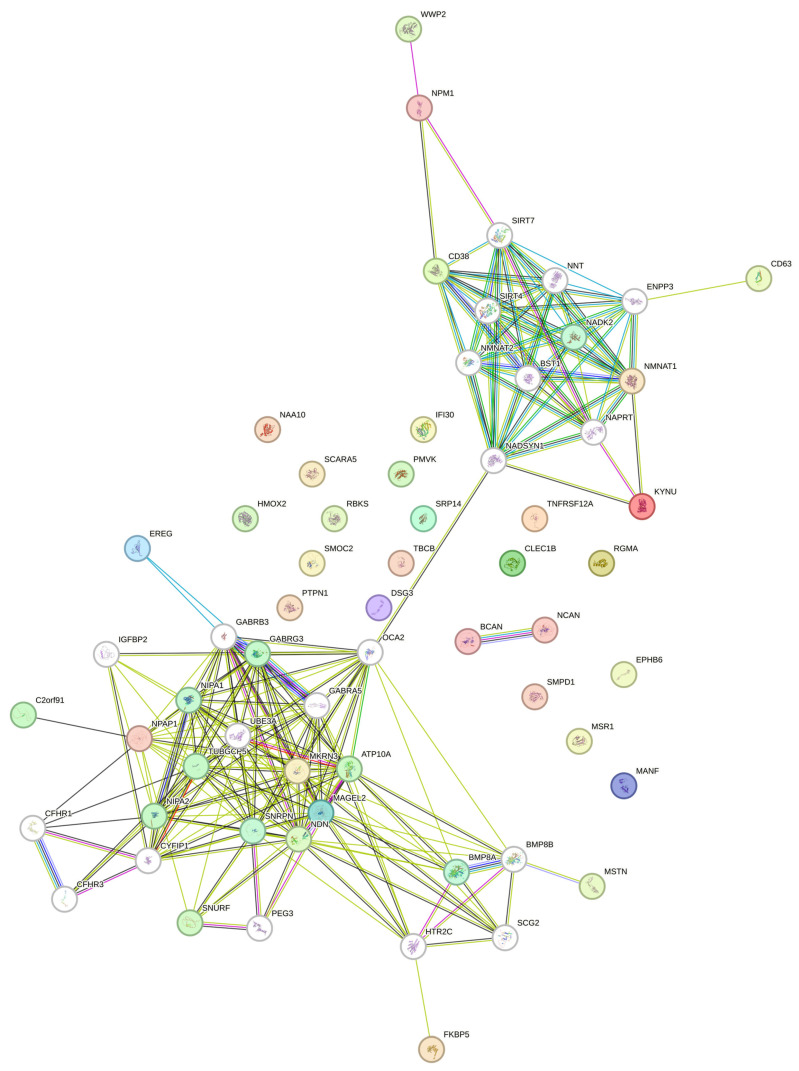
Protein–protein interaction network. The network was constructed in STRING Version 12 by using a gene list consisting of the 29 circulating proteins identified through the Olink profiling and the proteins encoded by the genes present in the PWS-critical region on chromosome 15, including small nuclear ribonucleoprotein (SNRPN), MAGE family member L2 (MAGEL2), Necdin-MAGE Family Member (NDN), Makorin ring finger protein 3 (MKRN3), and Nuclear pore associated protein 1 (NPAP1). White nodes indicate the second shell of interactors. Blue and purple lines indicate known protein interactions from curated databases and experimentally validated interactions, respectively. Green, red, and deep blue lines indicate predicted interactions based on gene neighborhood, gene fusions, and gen co-occurrence, respectively. Lime green, black, and lilac indicate other types of connections represented by text mining, co-expression, or protein homology, respectively.

We explored online protein–protein interaction databases to investigate whether genes within the PWS-critical region of chromosome 15 influence the expression of the circulating protein signature identified through circulating proteome analysis. Despite limited studies on genes within the PWS region, we identified a protein–protein interaction network highlighting a core group of proteins, including NPAP1, NDN, MKRN3, SRNPM, and MAGEL2. This core network is also connected to two distinct clusters of interacting proteins (Figure 3).

The first cluster includes Cluster of Differentiation 38, also known as Cyclic ADP-ribose Hydrolase 1 (CD38), Kynureninase (KYNU), WW domain-containing E3 ubiquitin protein ligase 2 (WWP2), Nucleophosmin 1 (NPM1), Nicotinamide nucleotide adenylyltransferase 1 (NMNAT1), and Tetraspanin-30/Ocular Melanoma-Associated Antigen (CD63) (Figure 3). The second cluster includes FKBP prolyl Isomerase 5 (FKBP5) and GDF-8/MSTN. These interactions underscore the complex network of protein interactions potentially involved in the pathophysiology of PWS, including other relevant proteins such as Ubiquitin-protein ligase E3A (UBE3A), Gamma-aminobutyric acid receptor subunit beta-3 and gamma-3 (GABRB3 and G3), the putative phospholipid-transporting ATPase VA (ATP10A), Gamma-tubulin complex component 5 (TUBGCP5), P protein (OCA2), and the magnesium transporters NIPA1 and 2 (Figure 3).

These findings suggest a possible, albeit indirect, effect of PWS-imprinted genes on some of the circulating proteins detected in our study. To further support this preliminary evidence, we delved into online datasets, as listed in Table 4, encompassing gene or protein expression data derived from the profiling of tissues or cells collected from PWS subjects or from animal and cellular experiments. Among the seven studies considered, only two partially corroborated our findings. Bochukova and colleagues [30] performed transcriptome analysis by comparing post-mortem hypothalamic tissue from four patients with PWS with BMI-matched subjects with non-syndromic obesity. Out of the 30 circulating proteins identified in our study, only CD63, FKBP5, N-Alpha-Acetyltransferase 10, NatA catalytic subunit (NAA10), PTPN1, and TNF receptor superfamily member 12A (TNFRSF12A) exhibited differential expression between the hypothalamic tissue of subjects with PWS and with non-syndromic obesity in the Bochulova’s study (Figure S1A and Table 4). The mRNA expression of these candidates was higher in the hypothalamus of subjects with PWS than those with non-syndromic obesity. At the same time, in our study, the circulating protein level was lower in the serum of subjects with PWS.

Another study, by Chen and colleagues [43], investigated the proteome of *Magel2+/+* (WT) mice and *Magel2p*−/*m*+ KO mice using a TMT-based approach across different tissues (Table 4). The Venn analysis revealed only two overlapping proteins among the datasets: Signal Recognition Particle 14 (SRP14) and Mesencephalic astrocyte-derived neurotrophic Factor (MANF) (Figure S1B,C). MANF was significantly downregulated in the liver of KO mice, while SRP14 was downregulated in the pituitary gland of KO mice. Interestingly, these two proteins were also downregulated in PWS subjects included in the present study. Notably, a poor overlap in the deregulated genes or proteins exists among studies (Table S2).

**Table 4:** List of profiling studies performed on subjects with PWS, animals, and cellular models of PWS.

Author	Study	Model	Source	Type of Analysis	Number of Differently Exp. Genes/Prot.	Number of Shared Deregulated Genes/Prot.
Bittel DC, 2007 [44]	Lymphoblastoid cells were established from 4 subjects with PWS with 15q11-q13 deletion, three subjects with PWS with UPD subjects, and three controls with non-syndromic obesity	Human	cells	Transcriptome	Top 50	none
Chen H, 2020 [43]	Tissues collected from *Magel2*p∆/m+ mice were analyzed through unbiased quantitative proteomic/mass spectrometry	Mouse	Hypothalamus	Proteome	263	none
			Brainstem	Proteome	254	none
			Adrenal gland	Proteome	37	none
			WAT	Proteome	34	none
			Liver	Proteome	457	MANF
			Pituitary	Proteome	184	SRP14
Bochukova EG, 2018 [30]	Through RNAseq, the post-mortem hypothalamic tissue of four subjects with PWS was compared with age and BMI-matched subjects with non-syndromic obesity	Human	Tissue	574	2980	CD63, FKBP5, NAA10, PTPN1, TNFRSF12A
Burnett LC, 2017 [45]	Induced pluripotent stem cell-derived (iPSC-derived) neurons from 2 subjects with PWS with large deletion (LD) and two subjects with PWS with microdeletion (MD), iPSC-derived neurons were also differentiated from 9 unaffected individuals	Human	Cells	Transcriptome (GSE89991 Analyzed by GEO2R)	100 (adj *p*-value)	none
Yazdi PG [46]	Skeletal (quadriceps) and whole brain samples from 3 mice with PWS-IC del and their WT age-matched littermates (*n* = 4) were analyzed by microarray	Mouse	Muscle	Transcriptome (GSE41759)	16 (adj *p*-value)	none
			Brain	Transcriptome (GSE41759)	24 (adj *p*-value)	none
Salles J, 2021 [47]	Seven subjects (five with deletions of the PWS locus, one with a microdeletion of *SNORD116*, and one with a frameshift mutation of *MAGEL2* with Schaaf–Yang syndrome), were compared to two control patients	Human	Blood	genome-wide methylation analysis	Top 50 hypomethylated and top 50 hypermethylated	none
Victor AK, 2023 [48]	Dental pulp stem cell (DPSC)-derived neurons from 4 neurotypical controls and 12 subjects with PWS (4 with deletion 8 with UPD)	Human	Cells	Transcriptome (GSE178687)	250 (adj *p*-value)	none

CD63: Tetraspanin-30/Ocular Melanoma-Associated Antigen; FKBP5: FKBP prolyl isomerase 5; MAGEL2: MAGE family member L2; MANF: Mesencephalic Astrocyte-derived Neurotrophic factor; NAA10: N-Alpha-Acetyltransferase 10, NatA catalytic subunit; PTPN1: Protein Tyrosine Phosphatase Non-Receptor Type 1; SRP14: Signal Recognition Particle 14; TNFRSF12A: TNF Receptor Superfamily member 12A; WAT: white adipose tissue.

### 3.5. Difference in the Circulating Protein Profiles Associated with Variations in the Metabolic Profile between the Two Groups

The circulating protein signature was evaluated using enrichment analysis based on the GO database, WikiPathways, KEGG, and Reactome, employing the g:Profiler tool (https://biit.cs.ut.ee/gprofiler/orth, accessed on 1 June 2024). Figure S2 illustrates the number of enriched pathways in each database. The differentially expressed circulating proteins were associated with several metabolic pathways such as ‘NAD metabolism’, ‘Tryptophan catabolism leading to NAD production’, and ‘NAD biosynthesis II from tryptophan’, involving KYNU, NMNAT1, and CD38 (File S2). Other significantly enriched pathways include ‘perisynaptic extracellular matrix’, ‘perineuronal net’, and ‘synapse-associated extracellular matrix’, in which BCAN and NCAN are involved (File S2).

When exploring DiGeNet for the association of the circulating proteins with diseases, among the top enriched diseases were ‘Hypertriglyceridemia’, ‘Diabetic wound’, and ‘Hyperinsulinism’ (Figure S3).

**Figure 4:**
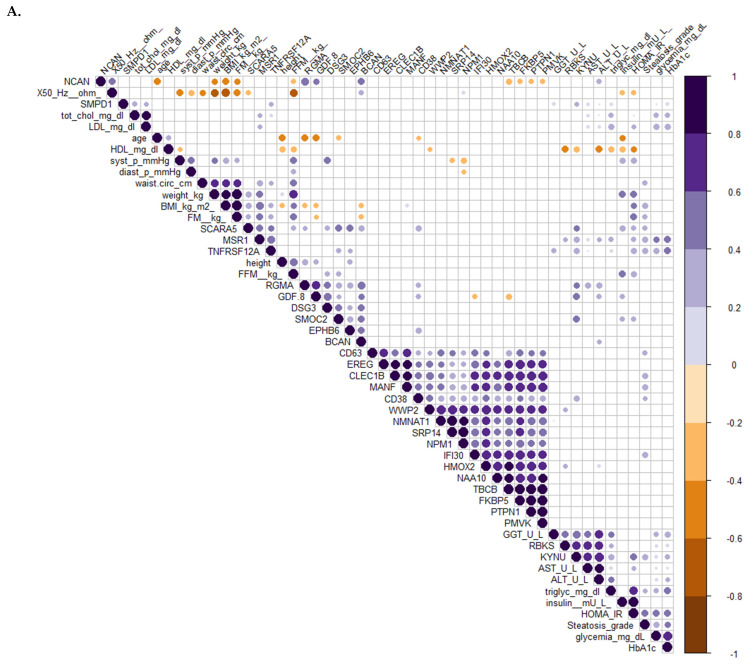

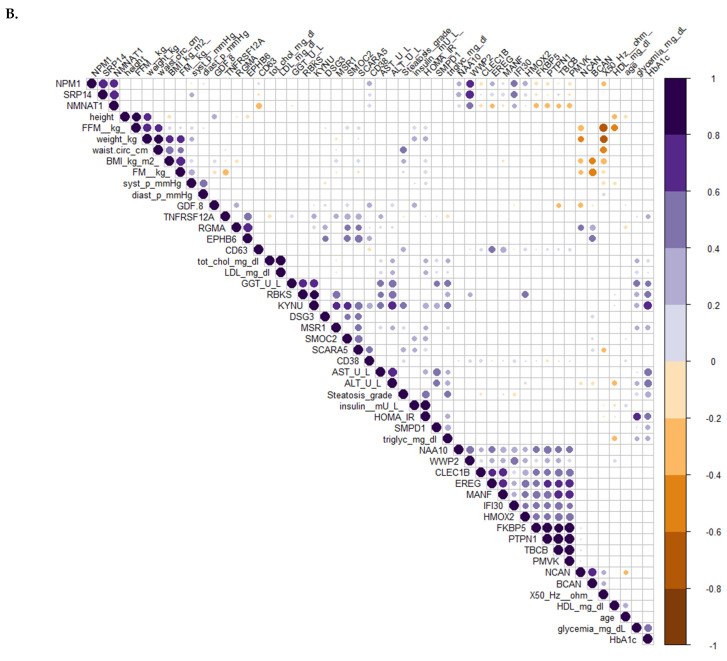
Clusterized correlogram of circulating protein candidates and clinical variables. Spearman’s correlation was used to determine the correlation coefficients. Unsupervised hierarchical clusterization was used to identify similar groups of correlating variables. The diameter and color depth of the dots are proportional to the p-value and correlation coefficient, respectively. Purple colors indicate a positive correlation while brown colors indicate a negative one. Only significant correlations were reported. (**A**) Correlation in PWS subjects. (**B**) Correlations in subjects with non-syndromic obesity.

Based on the correlations with clinical variables, the circulating biomarkers can be grouped into several clusters. Interestingly, correlation analysis in PWS significantly associated Ribokinase (RBKS), KYNU, Macrophage Scavenger Receptor 1 (MSR1), and SMPD1 with metabolic and liver dysfunction markers, such as transaminases, triglycerides, glycemia, and glycated hemoglobin (Figure 4A). Scavenger Receptor Class A Member 5 (SCARA5), TNFRSF12A, GDF-8, and Related Modular Calcium Binding 2 (SMOC2) were associated with insulin resistance markers and steatosis grade (Figure 4A). CD63, C-Type Lectin Domain Family 1 Member B (CLECB1), CD38, MANF, and Gamma-interferon-inducible lysosomal thiol reductase (IFI30) were all associated with steatosis grade in PWS (Figure 4A and Table S3). In OB, correlations between circulating protein markers and clinical variables were generally less evident, except for KYNU. MSR1, KYNU, and RBKS, but not SMPD1, were associated with metabolic and liver dysfunction markers (Figure 4B), while SCARA5, TNFRSF12A, SMOC2, but not GDF-8, were associated with insulin resistance markers. RBKS, but not SMPD1, was associated with metabolic and liver dysfunction markers (Figure 4B), while SCARA5, TNFRSF12A, and SMOC2, but not GDF-8, were associated with insulin resistance markers, and steatosis grade in the OB group (Figure 4B). In this group, CD63 and CD38 positively correlated with steatosis grade, while NCAN and BCAN positively correlated with bioimpedance values (Figure 4B and Table S3).

Altogether, these results illustrate the differences explaining the variations in the expression of the proteins and associated clinical variables observed between the two groups.

## 4. Discussion

Our exploratory study aimed to characterize circulating protein profiles in individuals with Prader–Willi Syndrome (PWS) and non-syndromic obesity. Due to the complexity of the disease and its poor molecular characterization, circulating proteome profiling assumes a particular relevance. It possibly aids in the identification of biomarkers specific to PWS, which can support diagnosis, monitor disease progression, and help tailor treatment strategies, potentially improving the management of PWS by addressing obesity and the neurological and psychological aspects of the syndrome.

We identified significant alterations in protein expression patterns between the two groups by utilizing a targeted proteomic approach based on the PEA technology. Those panels focus on markers associated with signal transduction, regulation of the nervous system, hormone release, and regulation of metabolism.

Notably, three proteins, DDR1, WFIKKN1, and GDF-8, also known as myostatin, exhibited significantly lower levels in female subjects with PWS than males, suggesting an association with neuromuscular impairment in female PWS subjects. Indeed, WFIKKN1 binds and inhibits GDF-8 [49,50,51], thus regulating muscle growth [52]. In previous studies, the serum levels of WFIKKNI were negatively correlated with aging [53]. However, this correlation was not observed in our PWS subjects.

GDF-8 serves as a negative regulator of muscle growth, and some studies suggested its potential role as a biomarker in neuromuscular disorders characterized by muscle atrophy and wasting. For instance, studies have linked reduced serum myostatin levels to the progression of genetic muscle disease [54], possibly attributed to the downregulation of the myostatin pathway as a compensatory mechanism in response to muscle wasting or atrophy [55]. Hypotonia, or decreased muscle tone, is a common characteristic of individuals with Prader–Willi syndrome regardless of gender [5]. Even if the severity of hypotonia can vary among individuals with PWS, to our knowledge, no studies have been conducted with the specific aim of identifying sex-related differences in this regard. However, it should be considered that GDF-8 may be influenced by multiple factors including diseases such as nutritional and metabolic status, inflammation [56], heart failure [56,57], and female fertility-related diseases [58]. Carvalho and colleagues, by studying a group of young adult obese patients, associated high GDF-8 and LP/ADP levels with metabolic syndrome, glucose-insulin homeostasis impairment, and low muscle mass [59]. Additionally, they found significantly higher GDF-8 serum levels in metabolically unhealthy females [59]. Considering the exploratory nature of the present study, it is crucial to extend the research on validating GDF-8 as a circulatory marker of hypotonia, especially in neonatal PWS subjects.

Our analysis of circulating proteome revealed significant differences in the expression levels of 29 proteins between individuals with PWS and subjects with non-syndromic obesity, with the majority of proteins displaying downregulation in the PWS group. However, several exceptions were noted, including BCAN, SMPD1, NCAN, DSG3, and EPHB6, which exhibited upregulation in PWS serum. Brevican and neurocan are extracellular matrix proteins primarily found in the central nervous system, contributing to neural development and synaptic plasticity [60,61]. Given their involvement in these critical processes, alterations in their expression levels may signify underlying neurological dysfunctions in PWS. Interestingly, previous studies have linked brevican and neurocan to various neurological diseases, including dementia, Alzheimer’s disease, epilepsy, and small vessel diseases [62,63,64]. The observed upregulation of brevican and neurocan in the serum of individuals with PWS raises intriguing questions regarding their potential roles in the pathophysiology of the syndrome. Further investigation is warranted to elucidate the specific mechanisms underlying their dysregulation and their implications for neurological function in PWS. Understanding the precise nature of the alterations associated with brevican and neurocan in PWS subjects could provide valuable insights into the neurological manifestations of the syndrome and potentially identify novel therapeutic targets. In this context, the decreased levels of RGMA, a glycosylphosphatidylinositol (GPI)-anchored protein that functions as a guidance molecule in the nervous system and which plays a crucial role in neural development, axonal growth, and cell migration [65], further underline the neurodevelopmental regulation impairment in PWS. Interestingly, a clinical case with moderate intellectual disability, epilepsy, and truncal obesity, suspected of PWS, showed a 15q26.1 microdeletion encompassing two genes: Chromodomain helicase DNA-binding protein 2 (CHD2) and RGMA [66].

Of particular interest is the serum reduction in CD38 observed in individuals with PWS. CD38, a type II transmembrane protein, plays a crucial role in regulating oxytocin release. Studies involving mice deficient in *Cd38* have shown decreased oxytocin levels in both plasma and cerebrospinal fluid, accompanied by elevated neuronal intracellular levels and abnormal social behavior [67]. The mechanism of CD38 in oxytocin release relies on cyclic ADP-ribose (cADPR) and nicotinic acid adenine dinucleotide phosphate (NAADP) [68]. This elucidates the enrichment observed in pathways related to ‘NAD metabolism’, ‘Tryptophan catabolism leading to NAD production’, and ‘NAD biosynthesis II’, influenced by other circulating proteins reduced in PWS, such as KYNU, an enzyme involved in Tryptophan metabolism [69,70] and NMNAT1, and denylyl transferase [71], both contributing to NAD metabolism. The protein–protein interaction network suggests the influence of genes within the PWS-critical region on a subset of genes or proteins associated with NAD-dependent oxytocin release. In particular, MAGEL2 inactivation has already been associated with alterations in the oxytocin system in mice [72,73]. Evidence suggests dysregulation of the oxytocin system in individuals with PWS, and these neuropeptide pathways may offer promising targets for therapeutic interventions, though the mechanisms underlying this dysregulation in PWS require further investigation [74]. Oxytocin-based therapies have been proposed, with studies indicating that low-dose intranasal oxytocin is safe for individuals with PWS and may lead to reductions in appetite drive, as well as improvements in socialization, anxiety, and repetitive behaviors [75]. Thus, there arises the possibility of using serum CD38 and oxytocin levels as guides for treatment strategies. The downregulation of genes involved in key metabolic pathways, such as KYNU, NMNAT1, and RBKS, underscores their significance in metabolic regulation. KYNU and NMNAT1 play roles in essential metabolic processes [76,77], while RBKS contributes to ribose metabolism [78]. In addition, the downregulation of MSR1 may impact both lipid uptake imbalance and/or neuroinflammatory pathways [79], potentially contributing to fat accumulation alterations on one side, and cognitive and behavioral challenges on the other, as observed in PWS [80,81]. These downregulations explain their positive correlation with clinical parameters associated with PWS, including LDL cholesterol, triglycerides, glucose levels, glycated hemoglobin, and HOMA index. These findings shed light on the complex interplay between genetic factors and the circulating protein signature identified in PWS, emphasizing the need for further investigation into the molecular mechanisms underlying this syndrome. Our findings align with previous studies, providing additional support for the potential impact of PWS-imprinted genes on circulating protein expression. Despite the variations in models and techniques used across existing studies, this consistency underscores the robustness of our observations. Nevertheless, further studies employing multi-omics approaches and functional validation experiments are warranted to validate the identified genetic connections and elucidate their precise roles in disease pathogenesis.

Despite the promising findings, this study has some limitations. One of the constraints is the relatively small sample size, both in terms of PWS cases and non-syndromic obesity controls. Moreover, the study’s exploratory nature should be considered when interpreting the findings. The aim was to generate hypotheses and provide a preliminary characterization of circulating protein profiles in PWS. As such, the results should be seen as a foundation for future research rather than definitive conclusions. Additional studies with larger cohorts are needed to validate the identified biomarkers and their potential role in clinics. In addition, we did not evaluate the influence of different comorbidities and medications on protein expression in the individuals. However, these factors were not the primary focus of the present project. Further studies are required to better understand these relevant aspects, which have been poorly investigated to date.

In conclusion, our exploratory study aimed to characterize circulating protein profiles in individuals with PWS and non-syndromic obesity. Given the complexity of PWS and the limited molecular characterization, circulating proteome profiling shows significant potential as a source of biomarkers for clinical purposes. In this context, the observed alterations in DDR1, WFIKKN1, and GDF-8 offer promise as gender-related biomarkers for neuromuscular impairment in female PWS subjects. Additionally, the changes in CD38 levels may indicate dysregulation of the oxytocin system in PWS, suggesting that monitoring its circulatory levels could guide oxytocin-based therapies. Future directions include longitudinal studies to track dynamic changes in circulating protein profiles and validation studies to establish the clinical utility of these biomarkers.

## Figures and Tables

**Table 1:** Clinical characteristics of the study groups.

Variable	OB (*n* = 34)	PWS (*n* = 53)	*p*-Value
Age (years)	35.2 ± 9.2	33.51 ± 10.9	0.45
Gender Female (*n*, %)	47%	55%	0.58
BMI (kg/m^2^)	41.1 ± 4.4	38.58 ± 8.94	0.13
Waist circumference (cm)	119.5 ± 14.3	120.87 ± 16.72	0.71
FFM (%)	54 ± 6.32	51.6 ± 7.2	0.12
FM (kg)	53.35 ± 8.73	44.62 ± 15.4	0.004
Systolic pressure (mm Hg)	129.4 ± 17.3	127.3 ± 9.6	0.46
Diastolic pressure (mm Hg)	80.6 ± 8.3	80 ± 6	0.69
Fasting glucose (mg/dL)	91.1 ± 14.8	101.7 ± 44.4	0.19
Insulin (mU/L)	19.8 ± 10.5	12.1 ± 7.31	0.00013
HOMA-IR	4.5 ± 2.7	3.1 ± 2.1	0.005
HbA1c (%)	5.5 ± 0.6	6 ± 1.6	0. 09
Total cholesterol (mg/dL)	184.8 ± 29.6	182.3 ± 36.5	0.74
HDL cholesterol (mg/dL)	43.9 ± 8.9	49.92 ± 14.2	0.03
LDL cholesterol (mg/dL)	124.3 ± 27.1	118 ± 29.5	0.32
Triglycerides (mg/dL)	140.6 ± 49.6	131.6.6 ± 100.72	0.63
AST (U.I./L)	23 ± 8.9	21.45 ± 11.2	0.51
ALT (U.I./L)	30.5 ± 16.5	28.7 ± 30	0.76
GGT (U.I./L)	42.8 ± 41.4	34.4 ± 58.7	0.47

BMI: body mass index; FFM: fat-free mass; FM: fat mass; HOMA-IR: homeostatic model assessment/insulin resistance; HbA1c: glycated hemoglobin; HDL: high-density lipoprotein; LDL: low-density lipoprotein; ALT: Alanine aminotransferase; AST: Aspartate aminotransferase; GGT gamma-glutamyl transferase; PWS: Prader–Willi syndrome; OB: non-syndromic obesity. Data are shown as the mean ± SD for continuous variables, and a number (%) for binary variables. A *t*-test was used to test for significant differences within continuous variables that were normally distributed, while Mann–Whitney and Kruskal–Wallis were used for non-normally distributed variables. The chi-square test was used for categorical variables. All of the statistical analyses were performed by GraphPad Prism 10.

**Table 2:** Proteins with significantly different abundance between female and male subjects with PWS.

Acronym	Protein	UNIPROT	M (*n* = 24)	F (*n* = 29)	Difference	FC	Adj. *p*-Value
DDR1	Epithelial discoidin domain-containing receptor 1	Q08345	8.59	8.35	−0.238	0.73	0.01
WFIKKN1	WAP, Kazal, immunoglobulin, Kunitz, and NTR domain-containing protein 1	Q96NZ8	4.70	4.19	−0.516	0.73	0.03
GDF-8	Growth/Differentiation Factor 8/Myostatin	O14793	3.93	3.21	−0.723	0.58	0.04

*p*-values were adjusted for multiple testing using the Benjamin–Hochberg method (adj. *p*-value). F: female; M: male; FC: linear fold of change.

**Table 3:** Proteins with significantly different abundances between PWS and OB subjects.

Acronym	Protein	UNIPROT	OB	PWS	Difference	FC	Adj. *p*-Val
RGMA	Repulsive Guidance Molecule BMP Co-Receptor A	Q96B86	11.5	11.1	−0.463	0.73	3.306 × 10^−5^
TNFRSF12A	TNF Receptor Superfamily Member 12A	Q9NP84	6.45	5.99	−0.461	0.73	0.0003438
GDF-8	Growth/Differentiation Factor 8/Myostatin	O14793	4.33	3.55	−0.788	0.58	0.0003438
MANF	Mesencephalic Astrocyte-Derived Neurotrophic Factor	P55145	9.22	8.8	−0.418	0.75	0.0003676
IFI30	Gamma-interferon-inducible lysosomal thiol reductase	P13284	5.67	5.08	−0.593	0.66	0.0007137
CLEC1B	C-Type Lectin Domain Family 1 Member B	Q9P126	12.5	12.1	−0.36	0.78	0.001707
BCAN	Brevican	Q96GW7	5.68	6.08	0.397	1.32	0.001707
HMOX2	Heme Oxygenase 2	P30519	2.36	1.97	−0.395	0.76	0.003391
TBCB	Tubulin Folding Cofactor B	Q99426	5.45	4.58	−0.872	0.55	0.003391
PMVK 0	Phosphomevalonate Kinase	Q15126	5.46	4.59	−0.869	0.55	0.003391
WWP2	WW Domain Containing E3 Ubiquitin Protein Ligase 2	O00308	5.48	5.11	−0.373	0.77	0.003391
EREG	Proepiregulin	O14944	5.19	4.34	−0.843	0.56	0.003391
NAA10	N-Alpha-Acetyltransferase 10, NatA Catalytic Subunit	P41227	4.02	3.51	−0.511	0.70	0.003391
PTPN1	Protein Tyrosine Phosphatase Non-Receptor Type 1	P18031	4.87	4.25	−0.627	0.65	0.005352
FKBP5	Peptidyl-prolyl cis-trans isomerase FKBP5	Q13451	6.53	5.69	−0.839	0.56	0.005503
MSR1	Macrophage Scavenger Receptor 1	P21757	7.46	7.09	−0.368	0.77	0.00734
NCAN	Neurocan	O14594	8.59	8.87	0.28	1.21	0.007448
KYNU	Kynureninase	Q16719	6.78	6.25	−0.529	0.69	0.01025
SCARA5	Scavenger Receptor Class A Member 5	Q6ZMJ2	8.02	7.84	−0.178	0.88	0.01025
CD38	Cyclic ADP-Ribose Hydrolase 1	P28907	5.36	5.07	−0.296	0.81	0.01025
EPHB6	Ephrin type-B receptor 6	O15197	7.46	7.73	0.266	1.20	0.01678
CD63	Tetraspanin-30/Ocular Melanoma-Associated Antigen	P08962	8.17	7.74	−0.424	0.75	0.01686
NMNAT1	Nicotinamide Nucleotide Adenylyltransferase 1	Q9HAN9	5.92	5.3	−0.626	0.65	0.01796
SMPD1	Sphingomyelin Phosphodiesterase 1	P17405	5.6	5.91	0.314	1.24	0.01796
SMOC2	SPARC Related Modular Calcium Binding 2	Q9H3U7	10.3	10	−0.256	0.84	0.02182
DSG3	Desmoglein 3	P32926	4.7	4.99	0.288	1.22	0.02272
NPM1	Nucleophosmin 1	P06748	4.45	4.02	−0.432	0.74	0.03768
SRP14	Signal Recognition Particle 14	P37108	5.78	5.27	−0.504	0.71	0.04092
RBKS	Ribokinase	Q9H477	7.84	7.44	−0.403	0.76	0.04723

*p*-values were adjusted for multiple testing using the Benjamin–Hochberg method (adj. *p*-value). PWS: Prader–Willi syndrome; OB: non-syndromic obesity, FC: linear fold of change in PWS vs. OB.

## Data Availability

Data are contained within the article and Supplementary Materials.

## Supplementary Materials

The following supporting information can be downloaded at: https://www.mdpi.com/article/10.3390/jcm13195697/s1, Figure S1: Venn diagrams of unique and shared genes/proteins; Figure S2: Protein enrichment analysis; Figure S3: Results of the protein enrichment analysis conducted in the DisGenNET database; Table S1: Main clinical features and medications; Table S2: Shared terms among different datasets; Table S3: Correlation clusters of the circulating protein biomarkers; File S1: List of protein targets included in the olink panels; File S2: Ranked list of the enriched pathways.
