# Supplementary material for: Characterization of Circulating Protein Profiles in Individuals with Prader–Willi Syndrome and Individuals with Non-Syndromic Obesity
Source: J Clin Med. 2024 Sep 25;13(19):5697. doi: 10.3390/jcm13195697 (PMC11476631; doi:10.3390/jcm13195697)
Supplement: Supplementary file 1 [file jcm-13-05697-s001.zip › Table s1.pdf]

Table S1: Main clinical features and medications

Clinical Features

	Feeding problems	Obstructive sleep apnea	Seizures	Hyperphagia	Delayed developmental milestones
PWS	53 (100%)	17 (32.1%)	1 (1.9%)	53 (100%)	51 (96.2%)
Non-syndromic obesity	0 (0%)	5 (14.7%)	0 (0%)	11 (32.4%)	0 (0%)

Medications

	Anti-hypertensives	Anti-diabetics	Anti-lipids	rec-GH	thyroxine	sex hormones	psychotropics
PWS	13 (24.5%)	17 (32.1%)	4 (7.5%)	17 (32.1%)	26 (49.1%)	26 (49.1%)	25 (47.2%)
Non-syndromic obesity	8 (23.5%)	6 (17.6%)	3 (8.8%)	0 (0%)	2 (5.9%)	3 (8.8%)	4 (11.8%)
