# Supplementary material for: Characterization of Circulating Protein Profiles in Individuals with Prader–Willi Syndrome and Individuals with Non-Syndromic Obesity
Source: J Clin Med. 2024 Sep 25;13(19):5697. doi: 10.3390/jcm13195697 (PMC11476631; doi:10.3390/jcm13195697)
Supplement: Supplementary file 1 [file jcm-13-05697-s001.zip › Table s2.pdf]

Table S2. Shared terms among different datasets

Studies	Number of shared terms	Genes/proteins
Bittel, Bochukova, Victor, Yazdi	1	SNRPN
Bittel, Burnett, Victor	1	IPW
Bochukova, Victor, Yazdi	2	SNHG14, NDN
Bochukova, Chen, Victor	1	TACC1
Bittel, Bochukova	4	OFD1, SDAD1, CNN3, FXYD3
Bittel, Chen	1	CDC5L
Bochukova, Burnett	10	CPXM2, SVOPL, PWAR6, GRIK4, HERC2, GALNT18, EMP3, SLC4A10, LRRC74B, CDC42BPB
Bochukova, Victor	40	PNMA1, IRS1, HERC6, CD93, PDXP,CTDP1, GADD45B, SERPINI1, SESN1, TIGD4, MPHOSPH9, DUS1L, DEPDC1B, KLF6, GJA4, ABCG2, DUSP6, DGAT2, MAGEL2, STC2, GDF15, S1PR2, IPO4, PWAR5, ZNF747, TRANK1, SNURF, OSGIN1, TFPI, TMEM115, GJA5, NMRK1, PIM1, MYO5A, SNTB2, JAG2, PHIP, AFAP1L2, LAMB1, JUNB
Bochukova, Salles	10	TFE3, CCDC110, EMD, DGKD, HIF3A, SMOX, WDR78, ABCG4, C1QTNF9, RPS7
Bochukova Chen	146	RPS17, KNDC1, CMPK2, COL1A1, WDR45, GFPT2, STAU2, C8G, RSPH4A, NB4, RBPMS, DCTN1, PI15, IGFBP2, CLIC5, NRSN1, RPLP, TRIP10, ALDH1L1, CSNK1E, PRMT5, CDC42EP5, ADH4, CA8, SHPK, SLC39A10, PDIA6, KCNJ16, RPL38, LPP, CCDC12, EPDR1, PRL, NIPSNAP1, GATAD2A, HAL, RPS13, MAPK9, RIPK2, MOCS3, CHL1, GBP2, C7, KHDRBS2, CAMK2G, MVP, WDR47, L1CAM, ASGR2, STXBP2, TIMM9, INA, MYCBP2, PABPC4, AKAP9, RPS19, FBL, EIF3L, RAB13, SLC12A5, BPHL, HSPH1, ACTB, AZIN2, PABPC1, SLC25A12, SV2C, ADAM22, AHNAK2, CRISPLD2, ANP32B, DPP10, TRAP1, AKAIN1, GOT1, CNN1, DNM1, FABP5, RPLP1, USP19, CRP, MGST1, ASAP2, TPT1, PXN, DDX39A, NAPB, ATP6V1A, FMOD, EEF1A2, INPP5F, CHGB, PFAS, EIF2B4, PLCB4, PIP4K2A, PRPF31, TNKS1BP1, MTFP1, EIF4EBP1, KRT7, GABRG2, ACACB, STX1B, NUDT4, ACTA1, TIMM17B, HSPA12A, AVP, ACOT1, MRI1, SLC2A13, EFHD1, NUDT7, SPTAN1, RPSA, RPS2, GABRB1, FBLN1, WARS, SARM1, KBTBD11, PPP2R2C, CTH, GBA, RRBP1, IL1RAP, CLASP2, TNC, CBFB, RACK1, NSF, ACADSB, SORBS1, AGRP, RPL23, ACTG2, CRTC3, CORO7, CISD1, TTPA, NME3, REXO2, ABCB8, NDRG3, MPST
Burnett, Victor	1	SNORD116-21
Burnett, Yazdi	1	ZMYND12
Burnett, Chen	3	NUBPL, DPP7, PDE4D
Salles, Victor	1	LYST
