# Supplementary material for: Characterization of Circulating Protein Profiles in Individuals with Prader–Willi Syndrome and Individuals with Non-Syndromic Obesity
Source: J Clin Med. 2024 Sep 25;13(19):5697. doi: 10.3390/jcm13195697 (PMC11476631; doi:10.3390/jcm13195697)
Supplement: Supplementary file 1 [file jcm-13-05697-s001.zip › Table S3.pdf]

Table S3. Correlation clusters of the circulating protein biomarkers

CLUSTERS	Correlations in PWS	Correlations in OB
**Metabolic and Liver Dysfunction**	MSR1, RBKS, KYNU, SMPD1	MSR1, KYNU, RBKS
**Insulin Resistance and Steatosis**	SCARA5, TNFRSF12A, SMOC2, GDF8	SCARA5, TNFRSF12A, SMOC2
**Liver Enzymes and Glycemia**	RGMA, BCAN, HMOX2	GDF-8, RGMA
**Steatosis Grade**	CD63, CLECB1, CD38, MANF, IFI30	CD63, CD38
**Negative Correlations with Age and Body Composition**	NCAN	
**Bioimpedance and Age**		NCAN, BCAN
**Blood Pressure and Bioimpedance**		NPM1, SRP14
**Lipid and Glycemic Markers**		SMPD1
