# Supplementary figures and images for: Characterization of Circulating Protein Profiles in Individuals with Prader–Willi Syndrome and Individuals with Non-Syndromic Obesity
Source: J Clin Med. 2024 Sep 25;13(19):5697. doi: 10.3390/jcm13195697 (PMC11476631; doi:10.3390/jcm13195697)

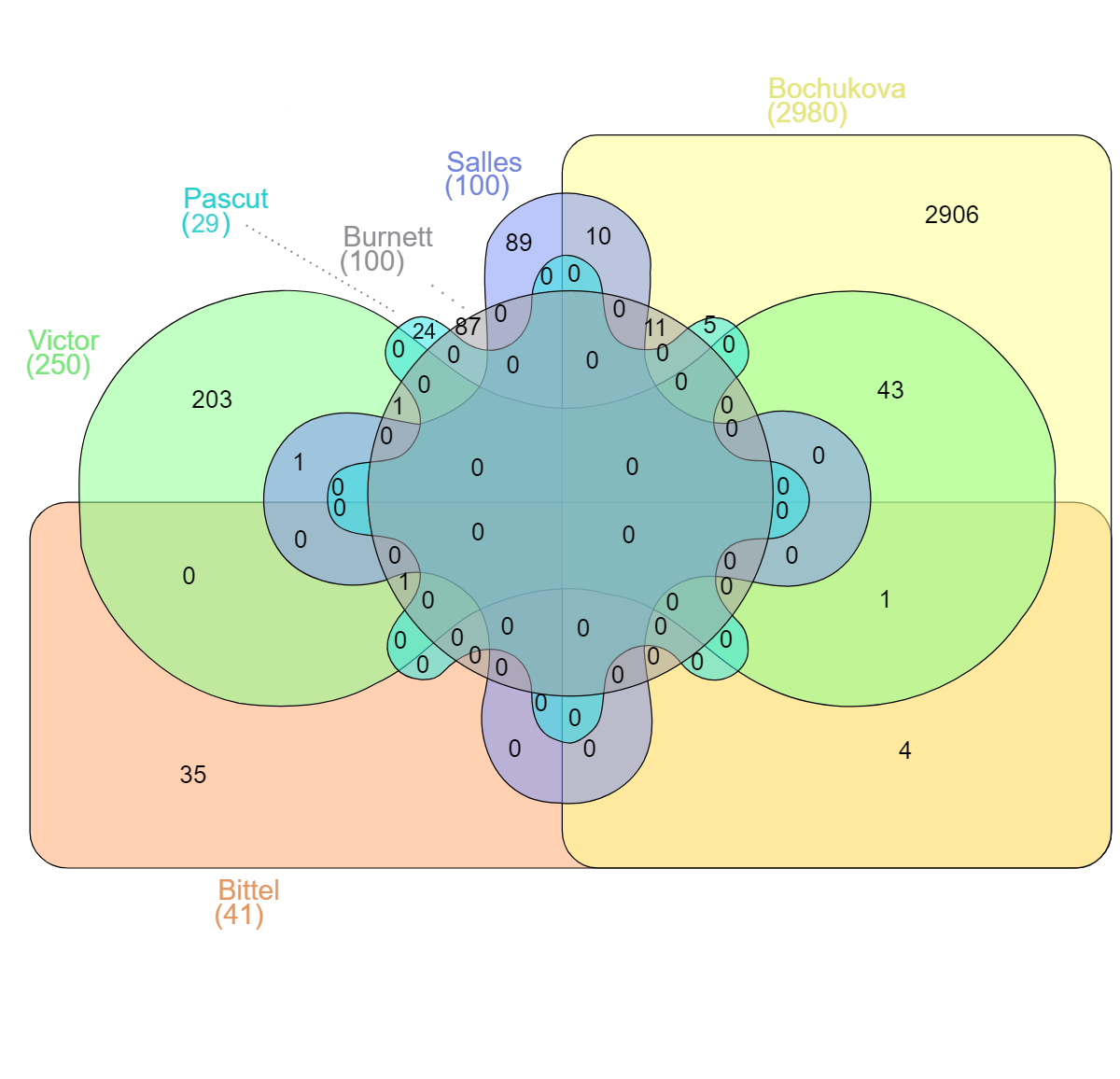

Supplement: Supplementary file 1 [file jcm-13-05697-s001.zip › Figure S1a.png]

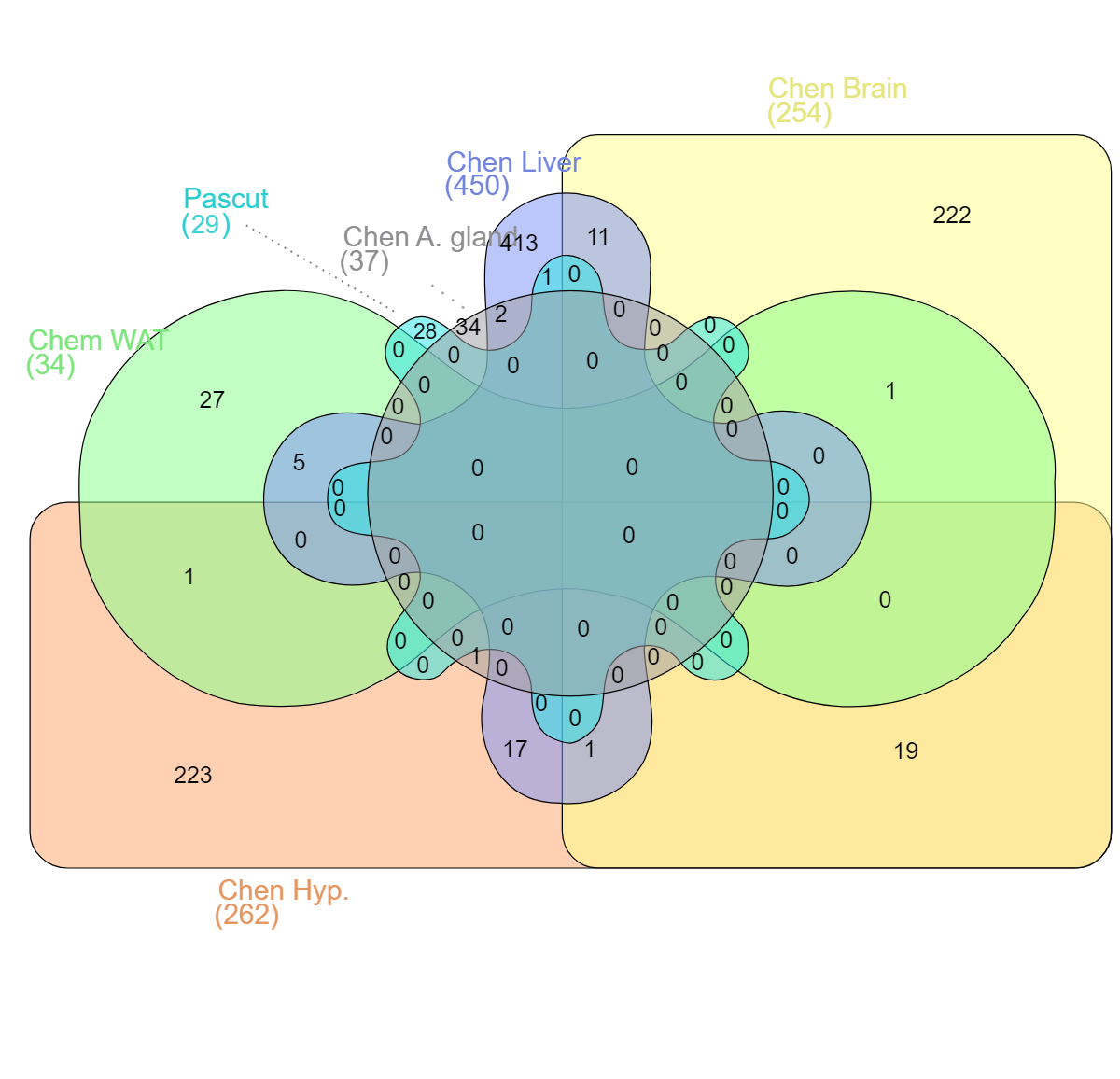

Supplement: Supplementary file 1 [file jcm-13-05697-s001.zip › Figure S1b.png]

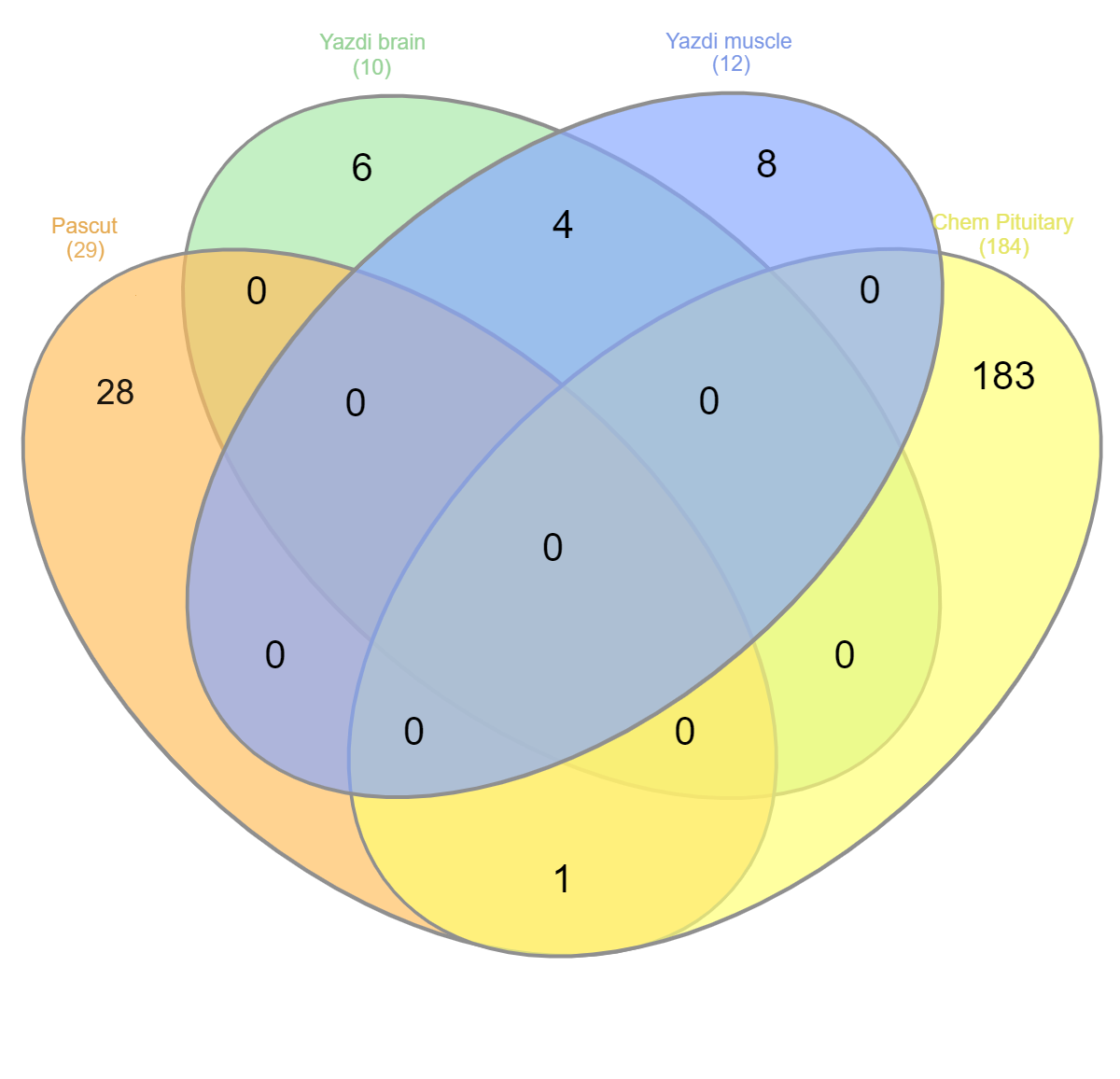

Supplement: Supplementary file 1 [file jcm-13-05697-s001.zip › Figure S1C.png]

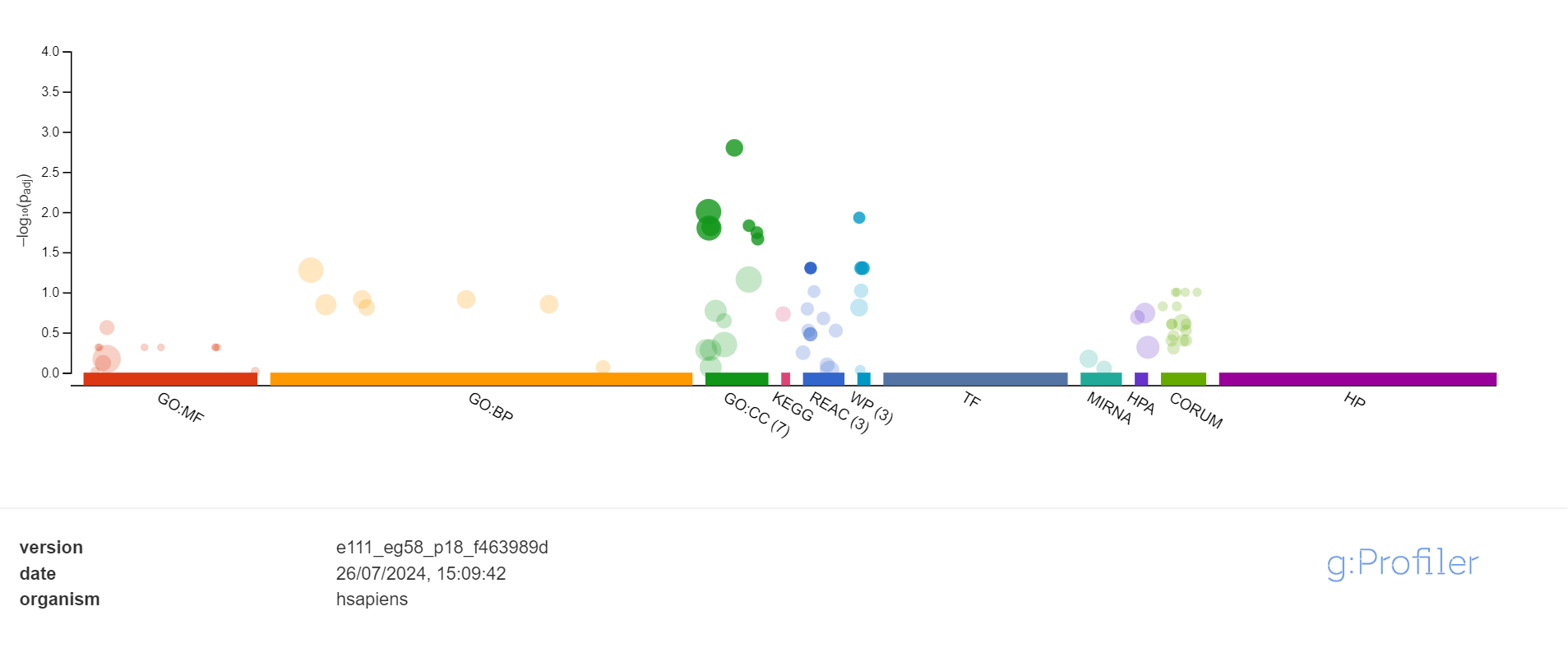

Supplement: Supplementary file 1 [file jcm-13-05697-s001.zip › Figure S2.png]

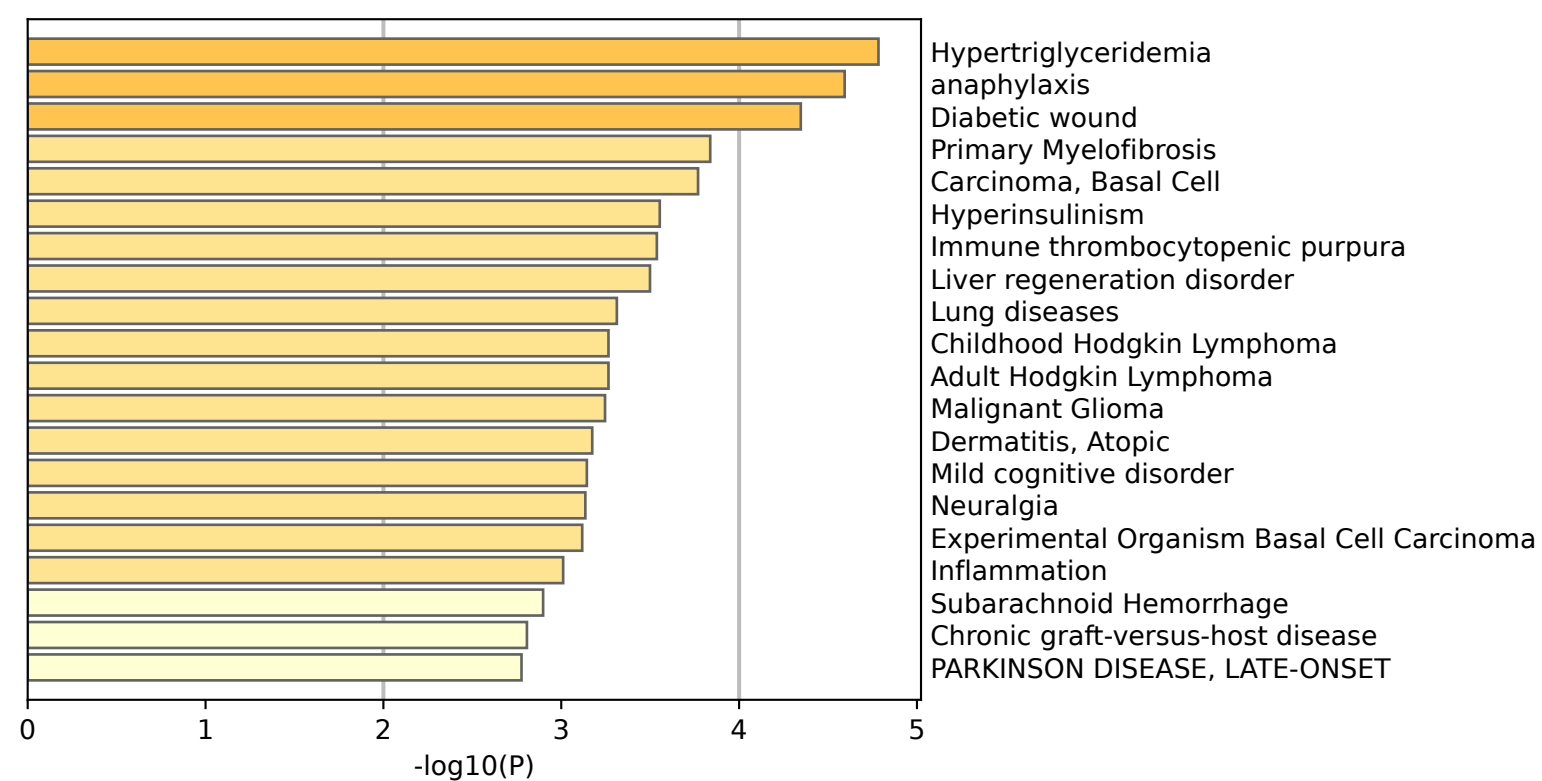

Supplement: Supplementary file 1 [file jcm-13-05697-s001.zip › Figure S3.pdf]
